# RETRACTION: Impact of Fast‐Track Rehabilitation Nursing on Pressure Ulcers and Postoperative Complications in Patients With Inter‐Trochanteric Fractures: A Meta‐Analysis

**DOI:** 10.1111/iwj.70324

**Published:** 2025-03-06

## Abstract

**RETRACTION:** ZhaiC. and LinY. , “Impact of Fast‐Track Rehabilitation Nursing on Pressure Ulcers and Postoperative Complications in Patients With Inter‐Trochanteric Fractures: A Meta‐Analysis,” International Wound Journal 21, no. 4 (2024): e14534, 10.1111/iwj.14534.38073014 PMC10961043

The above article, published online on 10 December 2023, in Wiley Online Library (http://onlinelibrary.wiley.com/), has been retracted by agreement between the journal Editor in Chief, Professor Keith Harding; and John Wiley & Sons Ltd. Following an investigation by the publisher, all parties have concluded that this article was accepted solely on the basis of a compromised peer review process. The editors have therefore decided to retract the article. The authors did not respond to our notice regarding the retraction.

**RETRACTION:** C. Zhai and Y. Lin , “Impact of Fast‐Track Rehabilitation Nursing on Pressure Ulcers and Postoperative Complications in Patients With Inter‐Trochanteric Fractures: A Meta‐Analysis,” International Wound Journal 21, no. 4 (2024): e14534, 10.1111/iwj.14534.38073014 PMC10961043

The above article, published online on 10 December 2023, in Wiley Online Library (http://onlinelibrary.wiley.com/), has been retracted by agreement between the journal Editor in Chief, Professor Keith Harding; and John Wiley & Sons Ltd. Following an investigation by the publisher, all parties have concluded that this article was accepted solely on the basis of a compromised peer review process. The editors have therefore decided to retract the article. The authors did not respond to our notice regarding the retraction.

